# Correction: Suppression of transcriptional drift extends *C. elegans* lifespan by postponing the onset of mortality

**DOI:** 10.7554/eLife.24506

**Published:** 2016-12-30

**Authors:** Sunitha Rangaraju, Gregory M Solis, Ryan C Thompson, Rafael L Gomez-Amaro, Leo Kurian, Sandra E Encalada, Alexander B Niculescu, Daniel R Salomon, Michael Petrascheck

We would like to thank David Angeles as he brought it to our attention that the variance equation (3) we provided in the original article was erroneous.

The equation in the published article read:

(3) $$dv=\frac{1}{n-1}+\sum_{i=1}^{n}\left({\text{td}}_{\text{i}}-\bar{td}\right)$$

It should have read:

(3) $$drift\;variance=\frac{1}{n-1}\sum_{i=1}^{n}{\left({\text{td}}_{\text{i}}-\bar{td}\right)}^{2}$$

We apologize for any confusion that may have occurred.

The article has been corrected accordingly.

